# 

**Published:** 1886-11

**Authors:** 


### CONTENTS.

ORIGINAL COMMUNICATIONS—

The Progress of Medicine. By Hunter P. Cooper, M. D., Atlanta, Ga. .... 529

Lectures on Diseases of the Skin. By Henry Wile, M. D., Atlanta, Ga.... 539

Two Unique Cases. By John Hockenhull, M. D., Cumming, Ga.... 546

Fistula in Perineo. By M. B. Hutchins, M. D., Lawrenceville, Ga.. 548

CLINIC REPORTS—

Acute Suppuration of the Middle Ear. By W. Cheatham, M. D., Louisville, Ky.... 550

SOCIETY REPORTS—

Chicago Medical Society.... 555

CORRESPONDENCE—

Our Philadelphia Letter.... 564

Our New York Letter.... 569

BOOK REVIEWS—

A Handbook on the Diseases of the Nervous System.... 572

Diseases of the Stomach and Intestines.... 573

A Manual of Dietetics.... 574

A Treatise on Electrolysis and its Applications to Therapeutical and Surgical Treatment in Diseases.... 575

Rheumatism—its Nature, its Pathology, and its Successful Treatment.... 576

Von Ziemssen's Handbook of General Therapeutics.... 577

A Manual of Practical Therapeutics.... 577

EDITORIAL—

An Anatomical Act for Georgia.... 579

The Office of Coroner.... 580

Yellow Fever Inoculation.... 581

Items.... 538, 549, 563, 582

Our Advertisers.... 592

